# Functional outcome after corrective osteotomy for malunion of the distal radius: a randomised, controlled, double-blind trial

**DOI:** 10.1007/s00264-020-04605-x

**Published:** 2020-05-29

**Authors:** Ingrid Andreasson, Gunilla Kjellby-Wendt, Monika Fagevik Olsén, Ylva Aurell, Michael Ullman, Jón Karlsson

**Affiliations:** 1 Department of Orthopaedics, Institute of Clinical Sciences, Sahlgrenska Academy, University of Gothenburg, Gothenburg, Sweden; 2 Department of Occupational Therapy and Physiotherapy, Sahlgrenska University Hospital, Mölndalsvägen 31, SE-431 80 Mölndal, Sweden; 3 Department of Physiotherapy, Institute of Neuroscience and Physiology, Sahlgrenska Academy, University of Gothenburg, Gothenburg, Sweden; 4 Department of Radiology, Sahlgrenska University Hospital, Gothenburg, Sweden; 5 Department of Radiology, Institute of Clinical Sciences, Sahlgrenska Academy, University of Gothenburg, Gothenburg, Sweden; 6 Department of Orthopaedics, Sahlgrenska University Hospital, Mölndal, Sweden

**Keywords:** Distal radius, Malunion, Corrective osteotomy, Subjective outcome, Grip strength, Range of motion

## Abstract

**Purpose:**

The purpose of this randomised, controlled, double-blind trial was to evaluate functional outcome during the first year after corrective osteotomy for malunited distal radius fractures, with or without filling the osteotomy void.

**Method:**

Patients were randomised to receive a HydroSet bone substitute or no graft. Cortical contact was maintained and stabilisation of the osteotomy was carried out with a DiPhos R- or RM Plate. To evaluate subjective functional outcome, the Patient-Rated Wrist Evaluation (PRWE), the Quick Disabilities of the Arm, Shoulder and Hand Questionnaire (Q-DASH), the Canadian Occupational Performance Measure (COPM) and the RAND-36 were used. Moreover, range of motion and grip strength were measured by blinded evaluators. Evaluations were made pre-operatively and three, six and 12 months post-operatively.

**Results:**

There were no significant differences between the groups at any time point post-operatively with respect to any of the PROMs that were used or range of motion or grip strength (*p* > 0.05). In both groups, there was a significant improvement at the 12-month follow-up compared with pre-operatively for the PRWE, the Q-DASH and the COPM satisfaction scores. The RAND-36 revealed no significant differences except for two domains, in which there was an improvement in the treatment group (*p* < 0.05). For grip strength and for range of motion in all movement directions, except dorsal extension, there was a significant improvement in both groups (*p* < 0.05).

**Conclusion:**

There is no significant difference in functional outcome during the first year after corrective open-wedge distal radius osteotomy, where cortical contact is maintained, regardless of whether or not bone substitute to fill the void is used.

## Introduction

Distal radius fracture is the most common injury in the orthopaedic emergency room [1]. A common complication of initially displaced and reduced distal radius fractures is healing with malunion [2]. This is reported to occur in approximately 35% of non-surgically treated fractures and up to 10% of surgically treated fractures [3, 4]. A malunion may cause pain and reduced range of motion (ROM) and may thereby hamper the ability to perform activities of daily living, take part in activities during leisure time or manage the demands of work [5, 6].

Corrective osteotomy is a surgical procedure that aims to ameliorate function by restoring the anatomy of the wrist. Various methods for performing this surgery have been described. Clinical symptoms, such as pain, reduced grip strength and reduced range of motion (ROM), rather than radiographic appearance, determine whether surgical intervention is indicated [7]. The procedure involves the re-creation of the fracture at or near the healed fracture site. The distal radius fragment is reduced until the radiographic appearance of the distal radius, the ulna and the carpal bones is normalised as effectively as possible [8]. Open-wedge osteotomy with plate fixation is regarded as the standard procedure for the common dorsally displaced Colles’ fracture [9]. It effectively restores the length of the radius, but, at the same time, it creates a void and grafts are often used to fill the void to create better stability [10]. An autograft from the iliac crest is often used, but this may lead to pain and other complications at the donor site [11]. To reduce complications of this kind, the use of a synthetic bone substitute is an option [12, 13], while leaving the void open is another option [14].

Previously, evaluations after different orthopaedic injuries, such as distal radius fractures, focused on radiological aspects and functional outcome in terms of grip strength and range of motion. In recent decades, the importance of taking the patients’ experiences and well-being into account, using different patient-rated outcome measurements (PROMs), has been emphasised [15]. This adds aspects that are likely to be of major relevance to the patients.

In a previous review of studies presenting results from surgery both with and without grafts, some of the studies included evaluations of subjective outcome, such as patient-rated wrist function and pain. This review indicated that the use of grafts was not necessary, either with respect to PROMs or with regard to regained function or radiographic outcome [16]. A recent, retrospective study, comparing the results of osteotomies, with or without the use of grafts, also indicates no differences in functional outcome between the methods [17]. Since corrective osteotomy is a relatively rare procedure, studies are often small, and more studies, including randomised, controlled studies, comparing functional outcome when a graft is or is not used, are needed.

The purpose of this study was to evaluate functional outcome during the first year after corrective osteotomy for malunited distal radius fractures, with or without filling the osteotomy void.

## Materials and methods

**Fig. 1:**
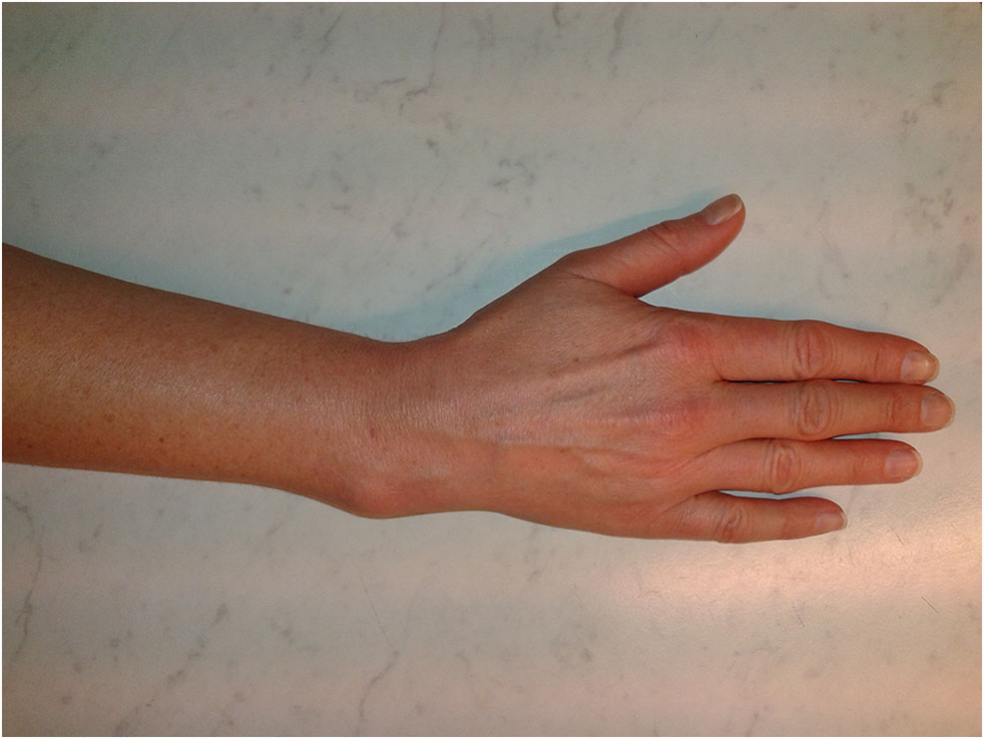
Image of the wrist of a woman aged 48 years suffering from a malunion of the distal radius

This randomised, controlled, double-blind trial comprised a series of patients undergoing osteotomy because of a malunited distal radius fracture between December 2014 and May 2018 (Fig. 1). The study was conducted on the Mölndal campus at Sahlgrenska University Hospital in Sweden. The indication for osteotomy was a malunion after a non-surgically treated distal radius fracture that was determined radiographically, in patients suffering from pain and functional limitations affecting their ability to perform activities of daily living.

The malunion warranted surgery if there was a dorsal tilt of > 20° with or without one or more of the following parameters:

- Radial inclination < 10°
- Radial shift > 5–10 mm
- Radial shortening > 5 mm

The exclusion criteria were dementia or not being able to communicate in Swedish without an interpreter. It was the surgeon who invited participants to the study after the pre-operative examination. After enrolment in the study, the patients visited the first author, who is an occupational therapist, and received both oral and written information about the study and the patients who chose to participate gave their informed, written consent. A total of 69 patients were assessed for eligibility, 11 did not meet the inclusion criteria, two were missed at inclusion, four declined to participate and eight decided not to undergo surgery. Recruitment started in December 2014 and follow-up was completed in May 2019. The study was performed in line with the principles of the Declaration of Helsinki and approved by the Ethics Committee in Gothenburg, Sweden (no. 472-14) and registered in a local register at Sahlgrenska Hospital (no. 29934). At the time the study began, it was not a prerequisite to register studies elsewhere.

The osteotomy was performed as a dorsal open-wedge distal radius osteotomy, which was fixed with a volar locking plate, using a two-incision procedure. The plate that was used was a DiPhos R Plate (Limacorporate**,** Udine, Italy), made of carbon fibre and PolyEtherEther Ketone polymer (PEEK), which is radiolucent [18, 19]. During the first year of the study, a narrower variant of the plate, DiPhos RM, was introduced to provide a better fit in patients with a thinner skeleton. The DiPhos RM Plate was used in four patients in the treatment group and in six patients in the control group.

The patients were randomised during surgery, by a person independent of the study, using the “Randomizer” app [20]. The ratio between the groups was 1:1. The treatment group had the osteotomy filled with HydroSet, a synthetic bone cement consisting of hydroxyapatite (HydroSet, Stryker Liebinger GmbH& Co. KG, D-79111 Freiburg, Germany), whereas the void was left empty in the control group.

### Post-operative regimen

Between four and seven days post-operatively, the patients were seen by an occupational therapist, or a physiotherapist. The patients were instructed to start performing exercises immediately, aimed at reducing post-operative oedema, every hour during the day. They were also told to use their hand in light daily activities and to keep it elevated during rest. The patients used a Softcast® fibreglass cast for two weeks. After two weeks, the cast was replaced by a brace (Wrist Lacer short 28571, Camp Scandinavia AB, Helsingborg, Sweden) and gentle range of motion exercises of the wrist were started four times a day according to a home exercise programme. All the patients followed this exercise programme and one also had supervised training at the clinic due to shoulder pain. The frequency of therapy sessions was individualised according to the patient’s status with regard to oedema and progress in range of motion during the treatment period. Patients were monitored either by therapists at the hospital or in primary care.

### Instruments

**Fig. 2:**
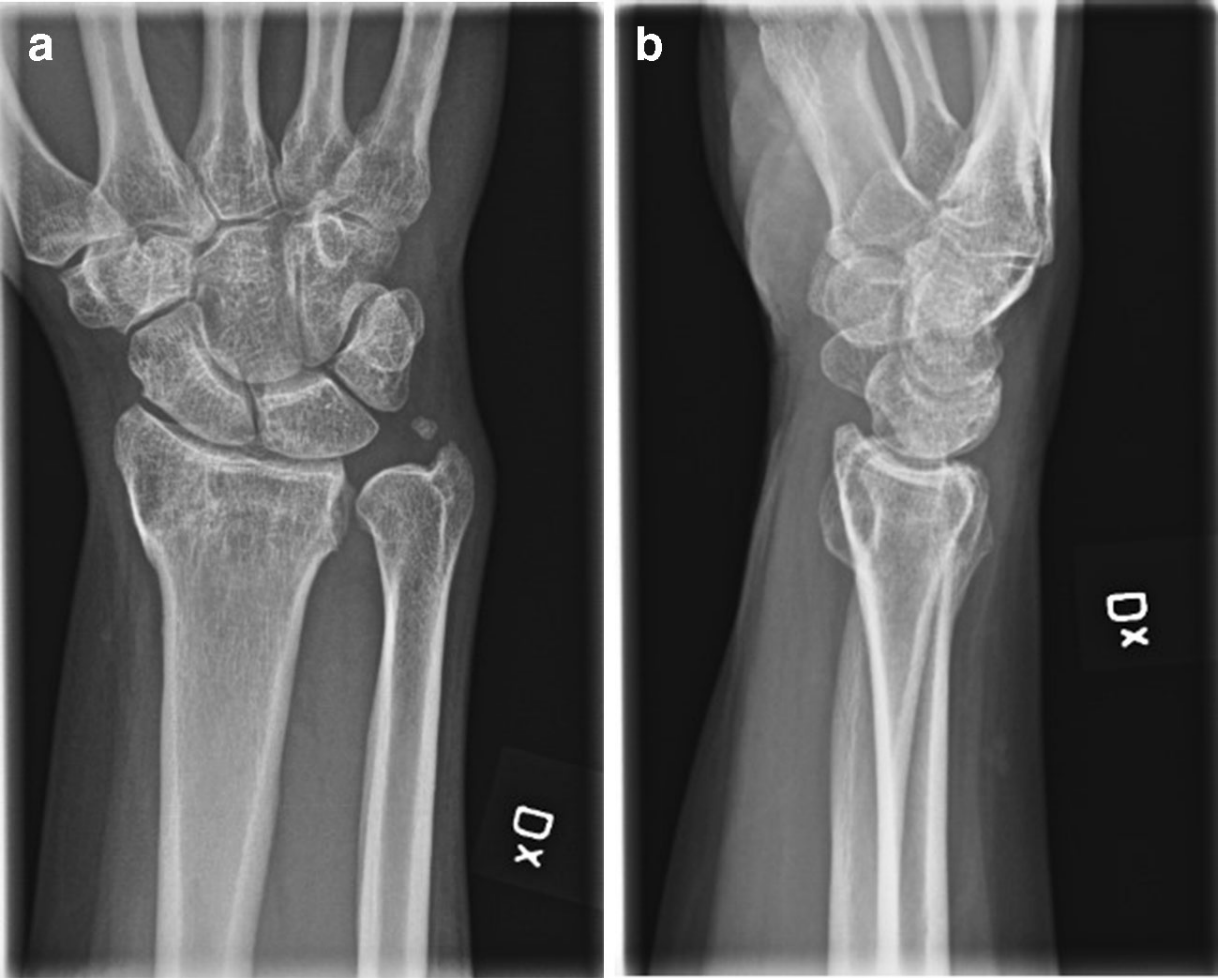
Pre-operative radiograph of malunited distal radial fracture scheduled for osteotomy with bone substitute. **a** AP view. **b** Sagittal view

**Fig. 3:**
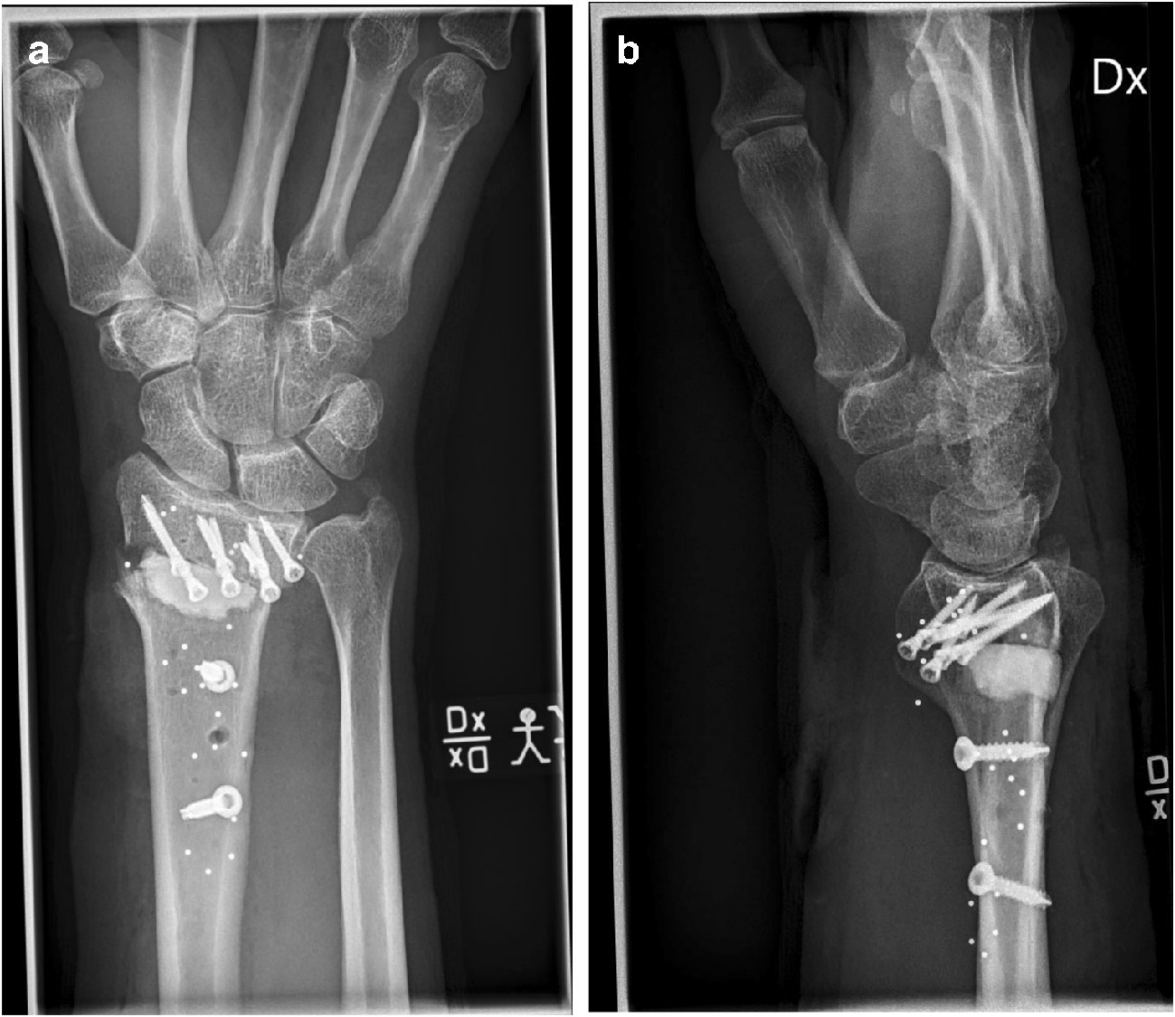
Post-operative radiographs after open osteotomy using bone substitute. **a** AP view. **b** Sagittal view

**Fig. 4:**
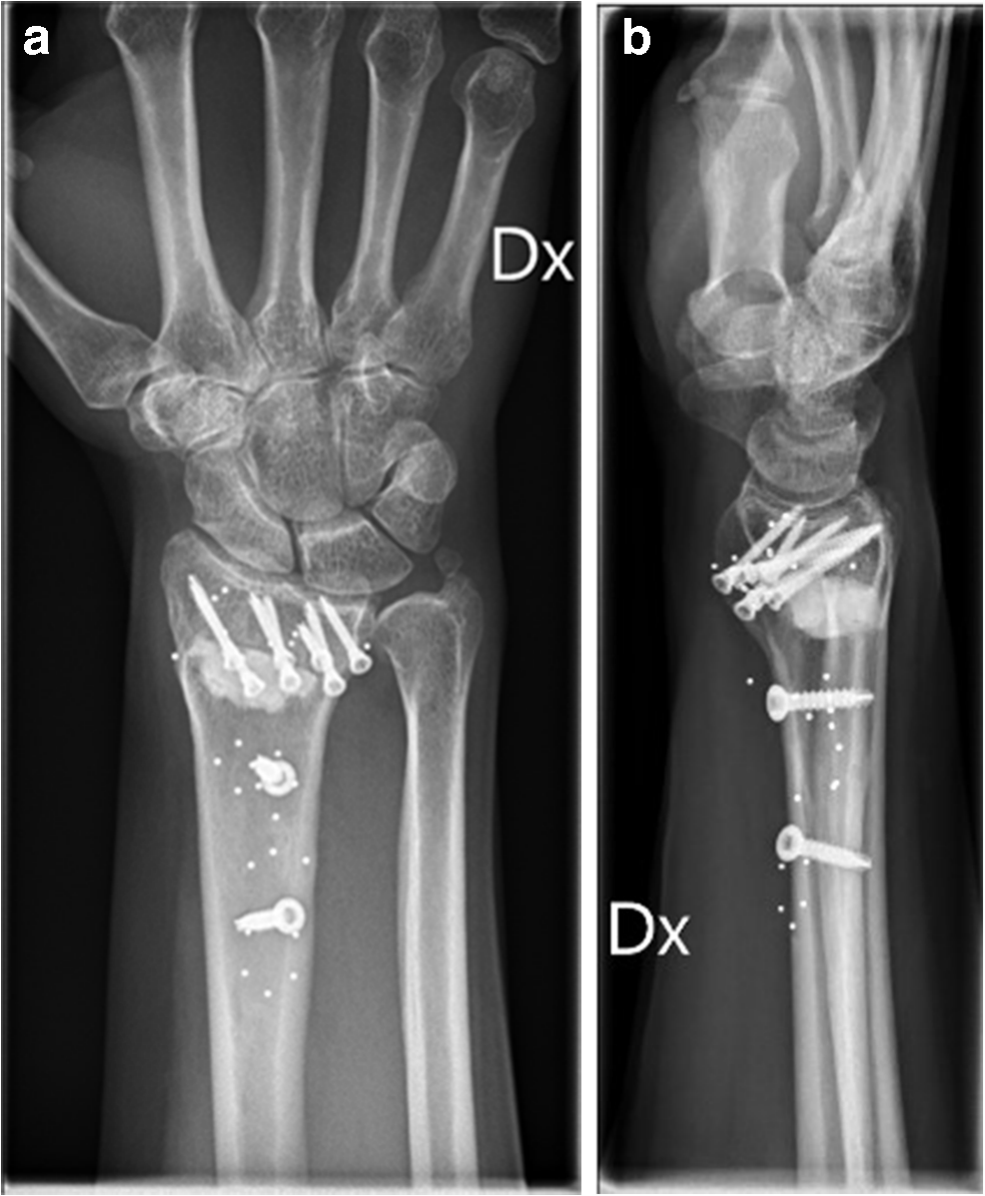
Radiographs at 12 months control after osteotomy using bone substitute. **a** AP view. **b** Sagittal view

The patients were seen pre-operatively and three, six and 12 months post-operatively to fill in different PROMs and for assessments of ROM and grip strength (Figs. 2, 3 and 4). With a few exceptions, the measurements were made by one occupational therapist. In these cases, two other specialised occupational therapists made the measurements in a standardised manner to ensure reliability. The occupational therapists were not engaged in the treatment of the patients and were blinded with respect to group allocation, as were the participants.

The following PROMs were used:

To measure the self-perceived ability to perform activities and pain:

- The Patient-Rated Wrist Evaluation (PRWE), which consists of five questions on pain and ten on function in specific activities, was used. The score ranges from 0 to 100 points and the higher the score, the poorer the function. The instrument has been tested for validity and sensitivity to change and is easy to fill in [21, 22]. The PRWE score was the primary outcome.
- The Quick Disabilities of the Arm, Shoulder and Hand (Q-DASH) questionnaire [23] was filled in pre-operatively and at the 12-month follow-up only. The Q-DASH consists of 11 items related to pain and function in the upper extremities. The score ranges from 0 to 100 points and the higher the score, the poorer the function [23]. The instrument has been tested for validity and reliability [24].
- The Canadian Occupational Performance Measure (COPM). This tool is used as a semi-structured interview, where the patient selects three to five activities or tasks of personal importance and assesses his/her own capability to perform these and how content he/she is with this ability [25]. The instrument has been tested for validity and reliability [26, 27].

To measure health-related quality of life:

- The RAND-36, which consists of 36 questions representing eight domains of quality of life, where a higher score indicates better health-related quality of life [28]. The instrument has been tested for reliability and responsiveness [29]. The RAND-36 was only filled in pre-operatively and at the 12-month follow-up.

Functional assessment:

- Grip strength was measured as the mean of three measurements using a Jamar dynamometer. The patient was in a seated position with the elbow in 90° of flexion [30].
- To measure the ROM, a goniometer was used [31] in a standardised manner according to a national measurement manual [32].

### Drop-outs

In the treatment group, one participant was lost to follow-up because of implant failure and a re-operation before the first follow-up and one was excluded from the analysis due to an implant failure within six months post-operatively. Both patients had the narrow plate. In the control group, one participant was lost to follow-up after an EPL rupture before the first follow-up. Two participants, with the original plate, were lost to follow-up due to implant failures and re-operation after falling on the operated arm before the first follow-up. One participant was excluded from the analysis due to the implant failure of the narrow plate within six months post-operatively. The patients who suffered implant failures between the first and the second follow-ups declined to undergo secondary surgery.

### Statistical analysis

We performed a sample size calculation based on the assumption that the group had a mean PRWE score of 40 points (SD ± 17 p) [33] pre-operatively and that the difference between groups should be 11 points, which is equal to the “minimal clinically important difference” (MCID) for patients with distal radius fractures [34]. To reach a power of 0.8 with an alpha of 0.05, 16 patients per group were needed. Forty-four patients were included to compensate for expected drop-outs.

The mean, SD, median and range were used to describe demographics. To compare the groups at baseline, the chi-square test was used for nominal data and the *t* test for numerical data.

The Mann Whitney *U* test was used to make comparisons between the groups on the follow-up occasions. Within-groups analysis was performed using Friedmann’s test and the Wilcoxon signed-rank test was conducted for comparisons within groups between follow-up occasions.

Analyses were performed with SPSS for Windows (Version 20, SPSS, Inc., Chicago, IL).

## Results

There were no significant differences between the patients completing the study, compared with the drop-outs in terms of age (*p* = 0.38), gender (*p* = 0.23) or injury to the dominant hand (*p* = 0.36).

The treatment group and the control group each comprised 15 women and four men.

The mean age in the treatment group was 59 (SD13) years and 55 (SD16) years in the control group. There was no statistically significant difference in terms of age between the groups (*p* = 0.32). In the treatment group, one patient, who was injured during childhood, underwent surgery 336 months (28 years) later. For the other patients, the range was 4 to 65 months from injury to surgery. Demographic data for both groups are shown in Table 1.

**Table 1:** Demographics

	Treatment group (*n* = 19)	Control group (*n* = 19)
Age at corrective osteotomy median [range]	59 [21–80]	65 [16–68]
Number of women	15 (79%)	15 (79%)
Number of men	4 (21%)	4 (21%)
Occupation at time of osteotomy		
Official	6 (32%)	4 (21%)
Labourer	5 (26%)	5 (26%)
Retired	6 (32%)	8 (42%)
Disability pension	2 (11%)	2 (11%)
Months to osteotomy median [range]	12 [4–336]	22 [6–65]
Injured limb		
Dominant	8 (42%)	8 (42%)
Non-dominant	11 (58%)	10 (53%)
Missing data		1 (5%)
Mechanism of injury		
Low energy	13 (68%)	12 (63%)
High energy	5 (26%)	7 (37%)
Missing data	1 (5%)	
Type of fracture		
Intraarticular	6 (32%)	9 (47%)
Extraarticular	13 (68%)	10 (53%)
Initial treatment		
Reduction	8 (42%)	7 (37%)
Type of implant		
DiPhos R	15 (79%)	13 (68%)
DiPhos RM	4 (21%)	6 (32%)
Complications		
EPL rupture	1 (5%)	1 (5%)
ECRL rupture	1 (5%)	
CTS	1 (5%)	
Need for plate removal		
DiPhos RM		1 (5%)
Implant failure		
DiPhos R		2 (11%)
DiPhos RM	2 (11%)	1 (5%)

### Comparison between groups

#### PROMs

There were no significant differences in self-perceived ability to perform activities and pain, measured with the PRWE, or in terms of activity performance measured with the COPM between the groups at any time point. Nor were there were any significant differences between the groups in terms of self-perceived ability to perform activities, measured with the Q-DASH, or health-related quality of life, measured with the RAND-36, pre-operatively or 12 months post-operatively (Tables 2 and 3).

**Table 2:** PRWE and COPM scores median (95% CI). Comparison between and within the groups, *p* value. There was no significant difference between the groups at any follow-up. *N* = 38

	Time point	Treatment group	Control group	*p* value
PRWE-total	Pre-operatively	58 (33–76)	58 (32–75)	0.9
	3 months	25 (9–44) •	26 (9–29) •	0.5
	6 months	24 (7–16)	23 (11–46)	0.8
	12 months	14 (7–31) ▪	20 (9–44) ▪	0.6
PRWE-pain	Pre-operatively	28 (21–41)	29 (21–33)	0.7
	3 months	12 (6–23) •	16 (9–22) •	0.3
	6 months	16 (10–44)	15 (9–22)	0.9
	12 months	9 (6–20) ▪	17 (6–24) ▪	0.3
COPM-performance	Pre-operatively	3 (2–4)	3 (3–5)	0.8
	3 months	5 (4–7) •	7 (4–9) •	0.4
	6 months	6 (4–9) •	8 (5–10) •	0.4
	12 months	10 (5–9)	9 (7–9) ▪	0.4
COPM-satisfaction	Pre-operatively	1 (1–4)	3 (1–4)	0.5
	3 months	6 (2–7) •	7 (4–9) •	0.4
	6 months	5 (5–9) •	6 (4–10) •	0.4
	12 months	8 (4–9) •▪	8 (6–9) ▪	0.8

• Significant difference compared with the previous test occasion *p* < 0.05

▪ Significant difference between values pre-operatively and 12 months post-operatively, *p* < 0.05

**Table 3:** RAND-36 and Q-DASH scores pre-operatively and at 12 months, median (95% CI), *p* value. Comparison between and within groups. *N* = 38

RAND-36	Treatment group		Control group		Comparison between groups	
	Pre-op	12 months	Pre-op	12 months	Pre-op	12 months
PF	65 (55–100)	80 (65–95)	70 (65–85)	80 (50–80)	*p* = 0.94	*p* = 0.99
RP	25 (0–50)	75 (25–100) ▪	50 (0–75)	50 (25–100)	*p* = 0.54	*p* = 0.56
BP	33 (23–55)	78 (35–90) ▪	45 (33–68)	55 (35–70)	*p* = 0.28	*p* = 0.19
GH	85 (60–90)	80 (70–95)	70 (60–85)	70 (50–90)	*p* = 0.33	*p* = 0.28
VT	70 (50–80)	70 (55–85)	70 (40–75)	70 (55–80)	*p* = 0.88	*p* = 0.51
SF	75 (63–100)	100(75–100)	75 (63–100)	100 (75–100)	*p* = 0.43	*p* = 0.68
RE	100 (67–100)	100 (67–100)	100 (33–100)	100 (67–100)	*p* = 0.53	*p* = 0.84
MH	80 (64–92)	88 (76–88)	84 (76–96)	84 (64–92)	*p* = 0.53	*p* = 0.43
Q-DASH	45 (25–55)	16 (5–27) ▪	36 (30–57)	20 (9–46) ▪	*p* = 0.8	*p* = 0.1

▪Significant difference between values pre-operatively and 12 months post-operatively, *p* < 0.05

#### Range of motion and grip strength

There were no significant differences between the treatment group and the control group with respect to range of motion or grip strength at any follow-up appointment (Table 4).

**Table 4:** Range of motion (degrees) and grip strength (Kg). Comparison between and within the groups. Mean (SD), *p* value. There was no significant difference between the groups at any follow-up. *N* = 38

	Time point	Treatment group	Control group	*p* value between groups
Supination	Non-injured side, pre-operatively	83 (7.3)	81 (7.3)	0.51
	Pre-operatively	67 (16.0) ♦	69 (10.1) ♦	0.76
	3 months	73 (11.5) •	73 (10.6) •	0.98
	6 months	75 (10.2) •	77 (8.9) •	0.30
	12 months	79 (8.9) •♦▪	78 (8.5) ▪	0.62
Pronation	Non-injured side, pre-operatively	73 (5.8)	72 (10.8)	0.49
	Pre-operatively	56 (17.7) ♦	58 (14.2) ♦	0.58
	3 months	63 (11.0) •	67 (10.0) •	0.50
	6 months	67 (9.7) •	70 (10.4) •	0.68
	12 months	68 (8.3) •♦▪	70 (10.3) ▪	0.98
Dorsal extension	Non-injured side, pre-operatively	70 (10.2)	72 (4.8)	0.23
	Pre-operatively	67 (14.2)	65 (15.2)	0.68
	3 months	63 (9.4)	63 (13.8)	0.99
	6 months	65 (9.5)	67 (12.4)	0.57
	12 months	66 (10.7)	67 (13.0)	0.77
Volar flexion	Non-injured side, pre-operatively	70.8 (10.2)	68 (11.5)	0.48
	Pre-operatively	41 (15.8) ♦	38 (16.4) ♦	0.62
	3 months	50 (11.0) •	47 (13.8) •	0.30
	6 months	52 (13.0)	52 (14.0) •	0.76
	12 months	54 (11.3) ♦▪	51 (14.6) ♦▪	0.41
Radial deviation	Non-injured side, pre-operatively	19 (6.1)	16 (5.1)	0.13
	Pre-operatively	15 (7.3) ♦	13 (6.7) ♦	0.55
	3 months	17 (5.2)	16 (6.2) •	0.74
	6 months	19 (4.7) •	17 (4.9)	0.36
	12 months	18 (4.8) ▪	17 (5.8) ▪	0.29
Ulnar deviation	Non-injured side, pre-operatively	27 (4.2)	27 (4.5)	0.96
	Pre-operatively	18 (7.1) ♦	20 (7.5) ♦	0.34
	3 months	21 (3.8)	22 (7.1) •	0.38
	6 months	23 (4.3)	25 (5.4) •	0.22
	12 months	24 (4.7) •♦▪	26 (4.7) ▪	0.21
Grip strength (kg)	Non-injured side, pre-operatively	31 (14.2)	30 (10.0)	0.74
	Pre-operatively	18 (14.4) ♦	20 (11.5) ♦	0.41
	3 months	24 (10.4) •	23 (8.1)	0.84
	6 months	26 (15.1)	26 (11.2)	0.84
	12 months	27 (9.2)	25 (6.5)	0.74

• Significant difference compared with the previous test occasion, *p* < 0.05

♦ Significant difference compared with the non-injured side, *p* < 0.05

▪ Significant difference between values pre-operatively and 12 months post-operatively, *p* < 0.05

### Comparison within groups

#### PROMs

There was a significant improvement at the 12-month follow-up, compared with pre-operatively, in the self-perceived ability to perform activities measured with the PRWE. Within the treatment group, the score decreased from a median score pre-operatively of 58 (95% CI 33–76) to 14 (95% CI 5–31) (*p* = 0.01), while the median score in the control group decreased from 58 (95% CI 32–75) to 20 (95% CI 9–44) (*p* = 0.01). There was also a significant improvement with respect to pain: the median score decreased from 28 (95% CI 21–41) to 9 (95% CI 2–20) (*p* = 0.001) in the treatment group and from 29 (95% CI 21–33) to 17 (95% CI 6–24) (*p* = 0.001) in the control group. The COPM showed a significant increase for both groups with regard to satisfaction but only for the control group in terms of activity performance (Table 2).

With respect to the Q-DASH score, there was a significant improvement in both groups. In terms of health-related quality of life, measured with the RAND-36, there was a significant improvement in the treatment group for the domain of “role physical” (RP) and the domain of “bodily pain” (BP). In the control group, there were no significant differences pre-operatively compared with 12 months post-operatively in any of the domains (Table 3).

#### Range of motion and grip strength

In both groups, ROM was significantly decreased in the injured side compared with the non-injured side pre-operatively for all movement directions (in both groups *p* < 0.05), with the exception of dorsal extension.

At 12 months post-operatively, there was a significant increase in both groups for pronation, supination, volar flexion and radial and ulnar deviation compared with pre-operatively (*p* < 0.05). There was no significant difference in either group regarding dorsal extension (*p* = n.s).

**Fig. 5:**
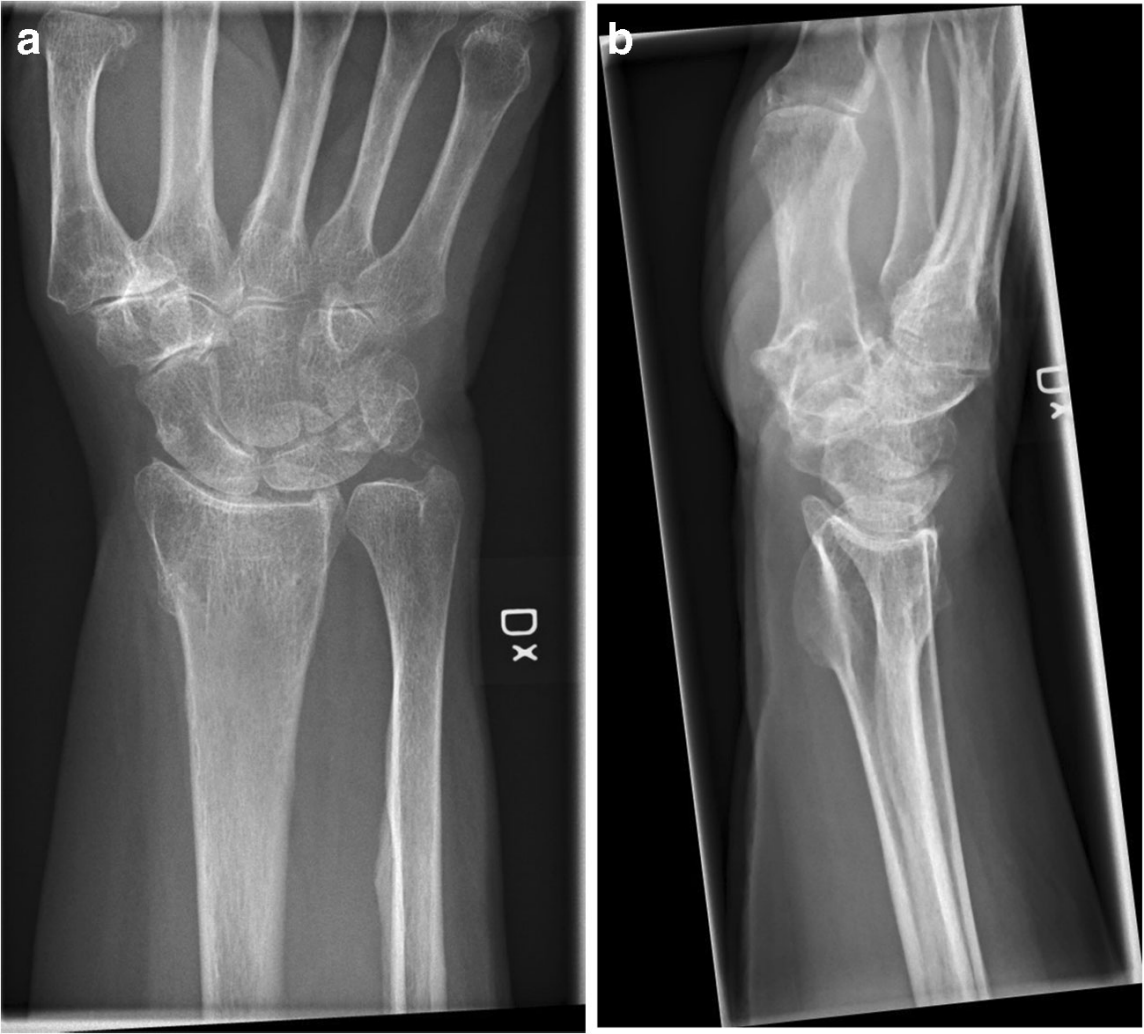
Pre-operative radiograph of malunited distal radial fracture. **a** AP view. **b** Sagittal view

**Fig. 6:**
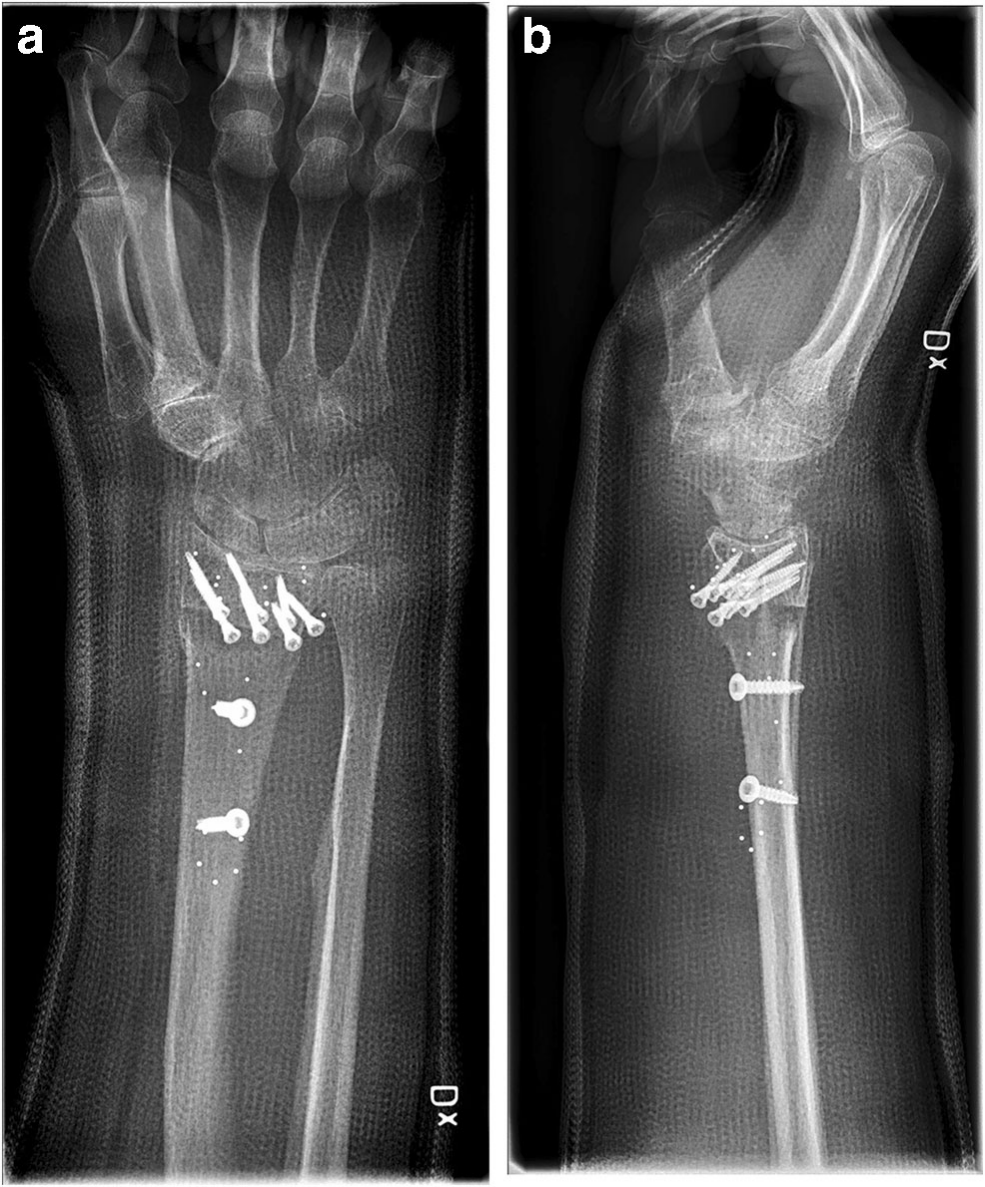
Post-operative radiographs after open wedge osteotomy. **a** AP view. **b** Sagittal view

**Fig. 7:**
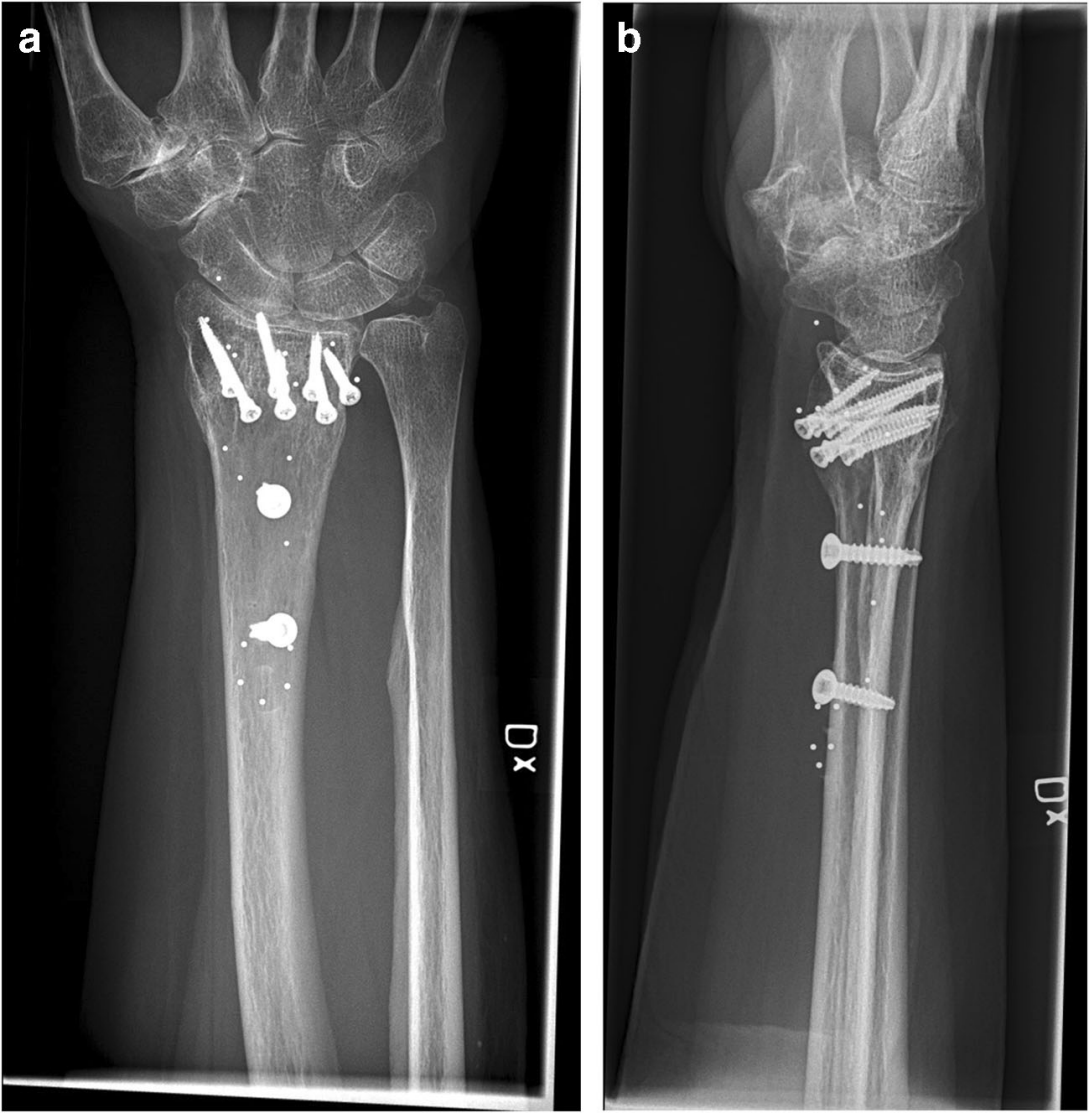
Radiographs at 12 months control after open wedge osteotomy. **a** AP view. **b** Sagittal view

When comparing the range of motion on the operated side with the non-injured side at the 12-month follow-up, the two groups differed. In the treatment group, there was still a significant difference between the non-injured side and the injured one, with respect to pronation, supination, volar flexion and ulnar deviation at the 12-month follow-up (*p* < 0.05). This indicates that the range of motion was not restored compared with the non-injured side. The control group experienced the restoration of ROM compared with the non-injured side in all directions apart from volar flexion, where there was still a significant difference compared with the non-injured side (*p* = 0.001) (Figs. 5, 6 and 7).

There was a significant increase in grip strength for both groups at 12 months compared with the pre-operative status in both groups (*p* < 0.05). In the treatment group, patients experienced the restoration of strength compared with the non-injured side at 12 months, as there was no longer a significant difference between the injured side and the non-injured side (*p* = 0.06). However, in the control group, the grip strength was still significantly decreased compared with the non-injured side at the 12-month follow-up (Table 4).

## Discussion

The results of this randomised, controlled, double-blind trial of 38 patients indicate that there were no significant differences in terms of functional outcome between the treatment group, in which the patients had the osteotomy void filled, and the control group, in which the void was left empty. There were no significant differences between the groups at any time point post-operatively in terms of any of the PROMs used, for either range of motion or grip strength.

The results of this study indicate that osteotomy after the malunion of a distal radius fracture improves self-perceived ability to perform activities, range of motion and grip strength. The functional outcome in this study is comparable with the results in another recent study comparing outcome for patients initially treated surgically and patients treated with reduction and a cast after comminuted distal radius fractures [35].

**Fig. 8:**
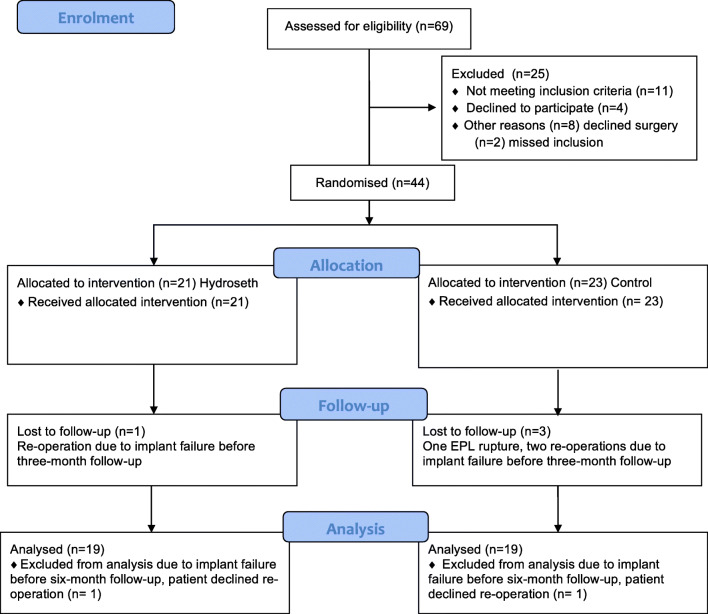
Flow diagram

The PRWE, the Q-DASH and COPM satisfaction scores had improved significantly 12 months post-operatively compared with pre-operatively for both groups. The minimal clinically important difference (MCID) can be defined as “the smallest change in an outcome perceived as beneficial by patients” [36–38]. The improvement in the PRWE score from a median of 58 points to 14 points in the treatment group and from 58 points to 20 points in the control group exceeded the MCID, which is 11 points in patients with distal radius fractures [34]. The PRWE pain score decreased from 28 (95% CI 21–41) to 9 (95% CI 6–20) (*p* < 0.05) in the treatment group and from 29 (95% CI 21–33) to 17 (95% CI 6–24) (*p* < 0.05) in the control group. These improvements also exceeded the MCID, which is 1.5 points for the PRWE pain scale, in patients with distal radius fractures [34]. Earlier research has shown that the functional outcome one year after a distal radius fracture is associated with a reduction in pain [39]. The results of this study indicate that, from the patient’s perspective, it is worthwhile undergoing surgery, since the improvement in both the PRWE scores and the PRWE pain scores was not only statistically significant but also exceeded the MCID 12 months post-surgery, compared with before (Fig. 8).

The results in terms of the RAND-36 indicate no differences pre-operatively to 12 months post-operatively, apart from an improvement in “role physical” and “bodily pain” in the treatment group. It is expected that health-related quality of life will not change that much in one year in this patient group, since they are not suffering from a progressive disease. An interpretation of the results is that the improvement in the wrist, which is reflected in the improvement in the PRWE and Q-DASH scores, is not reflected in the health-related quality of life in the way that it is evaluated using the RAND-36.

With respect to range of motion, the pattern of recovery was similar over the year in both groups. Dorsal extension was not significantly decreased compared with the non-injured side pre-operatively in either group, nor did it change significantly over the year. All other movement directions were significantly decreased in the injured wrist pre-operatively compared with the non-injured side. Volar flexion was the movement direction that was mostly decreased pre-operatively compared with the healthy side in both groups, and was the movement direction where the largest recovery was seen. However, also pre-operatively, both groups had enough capability in volar flexion to manage activities of daily living. About 40° of wrist flexion has been shown to be sufficient for activities in daily life [40]. The improvement in radial and ulnar deviation was probably too small to be noticeable for the patients. The changes in supination, pronation and volar flexion were large enough for the patients to detect. Taken one by one, the changes are probably not clinically important, but the sum of all the changes together is, as the total range of motion arc increased, enabling the wrist to function more effectively in everyday life activities. For the control group, the recovery in range of motion was so large that there were no longer any differences compared with the non-injured side in supination, pronation and radial and ulnar deviation. Moreover, in the treatment group, the recovery was also large enough for the patients to reach range of motion close to the values on the non-injured side and, again, there were no significant differences between the groups.

Concerning grip strength, there was a significant increase at the 12-month follow-up in both groups compared with pre-operatively. The change was large enough to presume that it was noticeable for the patients in both groups, which is clinically important, since the recovery of grip strength is important for the ability to perform activities of daily living [41]. The recovery, in terms of increased range of motion and grip strength, was reflected in the PROMs.

The results of this study indicate that the use of a bone substitute does not appear to imply any significant advantage as analysed by any of the PROMs used, or in terms of range of motion or grip strength. This finding is in line with the result of a recent study by Mugnai et al. [16], comparing the use of an iliac bone graft with a non-graft, with respect to time to healing and functional outcome. It revealed that osteotomies of extra-articular malunions do not require grafting when a volar locking plate is used [16]. Another recent study of 48 patients also revealed that there was no difference in functional outcome regardless of whether or not a graft was used [17]. Since there is a cost associated with using a bone substitute, surgeons should consider the possibility of not filling the void. In the present study, the osteotomies were carried out with the preservation of volar cortical contact, thereby facilitating healing. If osteotomy gaps are larger, a bone substitute or other grafts might, however, be necessary to maintain stability and facilitate bone healing.

There were some limitations to this study. During the time of the study, a narrower plate was introduced because the original plate appeared to be too wide to be suitable for patients with a thinner skeleton. The narrower plate was used in ten patients (four in the treatment group and six in the control group). It is possible that the change of plate affected the outcome of the trial. No calculations were made with respect to which kind of plate was used.

Another limitation is that five patients (two with the original plate in the control group and three with the narrower plate, of which two were in the treatment group and one in the control group) suffered an implant failure during the first year post-surgery. Because of the implant failure, they were excluded from the analysis.

In this trial, interest focused on functional outcome from the patient’s perspective. The results with respect to radiology, such as time to healing, remaining deformities and the occurrence of osteoporosis, are not presented or linked to functional outcome, which might be regarded as a limitation.

One strength in this study is that the surgery was performed by one surgeon. Other strengths are that the measurements were made by one, blinded, occupational therapist, with just a few exceptions when they were made by two other trained occupational therapists, and that the measurement methods and PROMs that were used have been tested for validity and reliability.

## Conclusion

There is no significant difference in functional outcome during the first year after corrective open-wedge distal radius osteotomy, where cortical contact is maintained, regardless of whether or not a bone substitute is used to fill the void.
